# Brain metastasis from thyroid adenomatous nodules or an encapsulated thyroid follicular tumor without capsular and vascular invasion: a case report

**DOI:** 10.4076/1757-1626-2-7180

**Published:** 2009-07-17

**Authors:** Tadashi Terada

**Affiliations:** Department of Pathology, Shizuoka City Shimizu HospitalShizuokaJapan

## Abstract

Because benign-appearing thyroid nodules with metastasis are very rare, the author reports herein four thyroid nodules (one follicular adenoma and three adenomatous nodules) with brain metastasis. A 75-year-old Japanese woman was admitted to our hospital because of thyroid mass. Imaging modalities revealed four distinct nodules in the thyroid, and tumorectomies of all nodules were performed under the clinical diagnosis of benign thyroid nodules. Grossly, one of them was a completely encapsulated tumor (35 mm in diameter). Other three nodules were non-encapsulated nodules (10 mm, 8 mm, and 7 mm in diameters). Multiple sections were obtained from the largest nodule. One section was obtained from each of the smaller three nodules. Microscopically, the largest tumor was an encapsulated follicular adenoma. The tumor consisted of normofollicles and microfollicles surrounded by a fibrous capsule. Neither capsular invasion nor vascular permeation was recognized. The parenchyma lacked nuclear atypia, mitotic figures, degenerative changes, papillary structures, nuclear inclusions, nuclear ground-glass features, and nuclear grooves. Thus, the largest tumor was diagnosed as follicular thyroid adenoma. The remaining three small nodules were typical adenomatous nodules composed of normofollicles and macrofollicles without nuclear atypia, mitotic figures, degenerative changes, papillary structures, nuclear inclusions, nuclear ground-glass features, and nuclear grooves. Therefore, a diagnosis of adenomatous nodules (goiters) was made. However, six years later, the patient showed a brain metastasis of thyroid tumor composed of macrofollicles without cellular and nuclear atypia. A diagnosis of metastatic follicular thyroid carcinoma was made. The present case suggests that benign thyroid nodules can metastasize.

## Introduction

Encapsulated thyroid follicular tumors without hstological evidence for carcinoma can metastasize^1^], and in the past such adenoma was called metastasizing adenoma 2-7. It has also been reported that thyroid adenomatous goiter can metastasize 8].

The thyroid neoplasms are now classified into follicular adenoma, follicular carcinoma, papillary carcinoma, poorly differentiated insular carcinoma, medullary carcinoma, anaplastic carcinoma, other neoplasms, and variant forms 3]. Papillary thyroid carcinoma tends to metastasize via lymphatic pathway, while follicular carcinoma via blood stream. The common metastatic sites are lymph nodes, lungs and bones, but brain metastasis is rare 10,11. The author herein reports a case of an encapsulated follicular tumor without capsular and vascular invasion and three adenomatous nodules (goiters) in the thyroid with a brain metastasis.

## Case presentation

A 75-year-old Japanese woman was admitted to our hospital because of thyroid mass. Imaging modalities revealed four clear cut nodules in the thyroid, and tumorectomies of the four nodules were performed under the clinical diagnosis of benign thyroid nodules.

Grossly, the submitted materials were the four thyroid nodules. One of them was completely encapsulated tumor (35 mm in diameter). Other three nodules were non-encapsulated nodules (10 mm, 8 mm, and 7 mm in diameters). No degenerative changes were found in the four nodules. Multiple sections were cut from the largest nodule. One section was obtained from each of the smaller three nodules. They were fixed in 10% formalin and embedded in paraffin wax. One 3-μm section was cut from each paraffin tissue block, and stained with hematoxylin and eosin.

**Figure 1.**
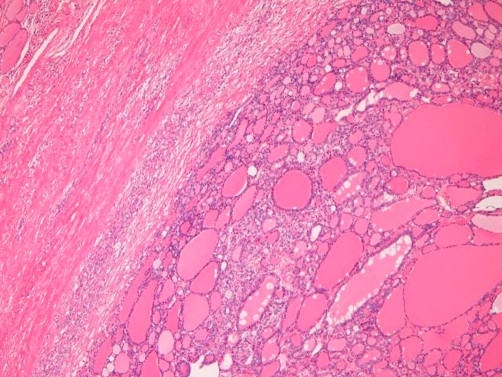
Follicular adenoma: The parenchyma is composed of normofollicles and microfollicles without atypia. A capsule is present (left). No capsular invasion or vascular invasion is recognized. HE, x40

Microscopically, the largest tumor was a completely encapsulated follicular adenoma. The tumor consisted of only normofollicles and microfollicles (Figure 1) surrounded by a fibrous capsule (Figure 1). Neither capsular invasion nor vascular permeation by tumor cells was recognized. The parenchyma lacked nuclear atypia, mitotic figures, degenerative changes, papillary structures, nuclear inclusions, nuclear ground-glass features, and nuclear grooves. Thus, the largest tumor was diagnosed as follicular adenoma. The remaining three small nodules were typical adenomatous nodules composed of normofollicles and macrofollicles without structural and nuclear atypia, mitosis, degenerative changes, papillary structures, nuclear inclusions, nuclear ground-glass features, and nuclear grooves. Therefore, the diagnosis was adenomatous nodules (goiters).

**Figure 2.**
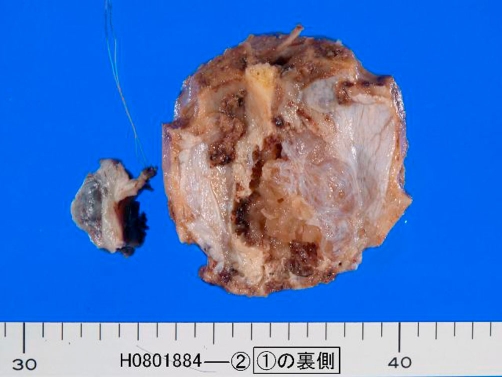
Gross features of brain tumor. It is a brown colored soft tumor.

**Figure 3.**
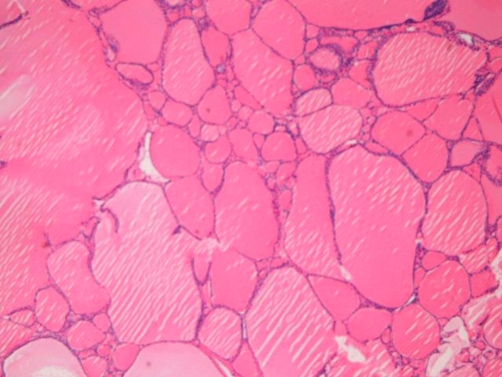
Brain tumor. It consists of macrofollicles without atypia. HE, x40.

However, the patient complained of headache six years later. Imaging (CT and MRI) revealed a brain tumor in the frontal lobe near the skull. A tumorectomy was performed under the clinical diagnosis of primary brain tumor. Grossly,the submitted material was a brown tumor measuring 6 x 6 x 4 cm(Figure 2). Four sections were obtained, and processed as mentioned above. Microscopically, the brain tumor was composed of macrofollicles without structural and nuclear atypia, mitotic figures, degenerative changes, papillary structures, nuclear inclusions, nuclear ground-glass features, and nuclear grooves (Figure 3). A diagnosis of metastatic follicular thyroid carcinoma was made.

## Discussion

The present case showed four thyroid benign-appearing nodules. However, the brain tumor developed six years later was metastatic follicular carcinoma. Therefore, one of the four nodules at the first operation was follicular thyroid carcinoma. The authors considers the following five possibilities: first the encapsulated thyroid follicular tumor was in fact an encapsulated microinvasive follicular carcinoma^12^]; second, one of the three adenomatous nodules was follicular carcinoma; thirdly, the remaining thyroid had follicular carcinoma; fourthly, the brain tumor arose from ectopic thyroid tissues in the brain,; and fifthly, the brain tumor is brain teratoma with an exclusive differentiation into thyroid tissue, similar to struma ovari.

Morphologies do not always reflect biological behaviors. The first hypothesis is most likely. The author extensively examined this encapsulated thyroid tumor, and could not find evidence of carcinoma. However, the examination employed only 3-μm thick HE sections of the paraffin tissue blocks. No step and serial section observations were performed. Therefore, it is possible that microinvasive area(s) of follicular carcinoma may be present in other sites but was not represented on the HE sections. A typical example supporting this hypothesis was reported by Mizukami et al. (1). They reported a case of an encapsulated follicular thyroid carcinoma without capsular and vascular invasion with bone metastasis. Their case is 42-year-old man who had histologically typical follicular thyroid adenoma without vascular and capsular invasion (1). The tumor was completely encapsulated and composed of microfollicles free of nuclear atypia and mitotic figures (1). However, the tumor metastasized to bone 22 years after a thyroidectomy. The largest encapsulated follicular tumor of the present case is very similar to that of Mizukami et al. (1). Taken together, it can be concluded that an encapsulated follicular tumor without capsular and vascular invasion can metastasize.

The second hypothesis is also likely, because it is recognized by Ito et al 8] that benign thyroid nodule including adenomatous nodule can metastasize. They reported that five (0.17%) of 2,975 adenomatous thyroid nodules without pathological evidence of carcinoma metastasized to lymph nodes and distant organs, and insisted that pathological diagnosis of thyroid nodules has limitations and cases diagnosed as benign adenomatous nodule should undergo careful follow-up (8). It is possible that the three thyroid adenomatous nodules in the present case metastasized to the brain.

The third hypothesis is also likely, because scrutiny of the remaining thyroid after tumorectomy was not performed. The fourth hypothesis is unlikely, because ectopic thyroid in the brain has not been reported, to the best of author's knowledge. The fifth hypothesis is possible, but the probability is very low.

In any way, thyroid nodules are protean. Many sections should be taken from the thyroid lesions, and step and serial sections should be done if possible.

However, pathological examination is limited because morphologies do not always reflect the biologic behaviors in thyroid lesions.

In summary, the author reported four benign-appearing thyroid nodules with brain metastasis.

## List of abbreviations

CT: computed tomography
MRI: magnetic resonance imaging

## References

[bib-001] Mizukami Y, Nonomura A, Hayashi Y, Ohmura K, Michigishi T, Noguchi M, Nakamura S, Ishizaki T (1996). Late bone metastasis from an encapsulated follicular carcinoma of the thyroid without capsular and vascular invasion. Pathol Int.

[bib-002] Nigri G, Berardi T (1969). Metastasizing adenoma of the thyroid gland. Acta Chir Ital.

[bib-003] Lorenz G (1972). Metastasizing thyroid adenoma. Zentralbl Chir.

[bib-004] Siewert R, Becker HD, Peiper HJ (1972). Metastasizing thyroid adenoma. Chirurg.

[bib-005] Kashigina EA, Girshin SD, Guteman GM, Lebedev MI (1980). Metastasizing adenoma of the thyroid. Vestn Rentgenol Radiol.

[bib-006] Yucel M, Muller E, Czerny R (1980). The so-called metastasizing adenoma from the orthopaedic view point. Z Orthop Ihle Grenxgab.

[bib-007] Ehrich JC, Kaneko M (1957). Metastasizing adenoma of the thyroid gland: a brief reconsideration with report of two cases. J Mt Sinai Hosp NY.

[bib-008] Ito Y, Yabuta T, Hirokawa M, Fukushima M, Inoue H, Urano T, Kihara M, Higashiyama T, Takamura Y, Miya A, Kobayashi K, Matsuzaka F, Amino N, Miyauchi A (2008). Distant and lymph nodes metastases of thyroid nodules with no pathological evidence of malignancy: a limitation of pathological examination. Endocrine J.

[bib-009] Rosai J, Carcangiu ML, DeLellis RA (1992). AFIP Atlas of tumor pathology. Tumors of the thyroid gland.

[bib-010] Yousuf K, Archibald SD (2006). Brain metastasis of papillary carcinoma of the thyroid. J Otolaryngol.

[bib-011] Simon N, Quyyumi SA, Rothman JG (2004). Follicular thyroid carcinoma presenting as a sellar mass: case report and review of the literature. Endocr Pract.

[bib-012] Thompson LD, Wieneke JA, Paal E, Frommelt RA, Adair CF, Heffness CS (2001). A clinicopathologic study of minimally invasive follicular carcinoma of the thyroid gland with a review of the English literature. Cancer.

